# Pneumonitis*The term “pneumonitis” is employed, rather than pneumonia, because it more definitely implies the presence of a localized pathological process as well as a universal febrile excitation of the corpuscular, cellular, proto­plasmic, and atomic elements of the organism.

**Published:** 1911-11

**Authors:** 


For Texas Medical Journal.

### "Pneumonitis."*

Under the above heading Dr. Robert Lee Hammond of Woodsboro, Maryland, U. S. A., has contributed an elaborate paper upon this intensely interesting subject.

The theme is unique in that it *systematically* and specifically impels the febrile movement as well as the inflammatory process to end by "crisis," which is as its nature would have it, rather than by "lysis."

The "extremities"—"hands, feet, head and nape of neck"—are repeatedly and thoroughly "wet" or "douched" with "ice water" and constantly and vigorously "fanned" with the result of aiding the evaporation of the water, and effecting the rapid radiation and carrying away of the heat from the nerve centers.

Hot and cold measures vie with each other as antipyretic and stimulating agents.

An "ice cap" also is applied to the praecordium *when found necessary.*

And these means failing to impel—rather than compel—for it is a conservative and not essentially a forceful method of treatment—

*The term "pneumonitis" is employed, rather than pneumonia, because it more definitely implies the presence of a localized pathological process as well as a universal febrile excitation of the corpuscular, cellular, protoplasmic, and atomic elements of the organism.

the advent of the "crisis," as a dernier expedient, a large, strong "cantharidal blister" is applied to the praecordium overlapping the lower region of the thorax.

*The dependable medicinal antipyretic, antiseptic, anodyne, analgesic, calmative, and stimulating agents, play their indispensable parts.*

Particular attention is given to the proper nourishment with the important object in view of maintaining a reserve strength, available for immediate use at critical periods, in the management of the case.

Daily "lavage" of the intestines with a three-pint normal saline solution as hot as can be borne is urged, unless the salt is specifically contra-indicated, then sterilized water with the addition of a tablespoonful of glycerine to each pint of water is used in its stead.

The temperature of the room must of necessity vary in the treatment, but it is kept at or as near 70 degrees Fahr. as possible during the application of the refrigerative treatment; but at the time of the "crisis" and while the hot applications are being used, with the object of stimulating the patient or relieving shock or an asthenic condition, the temperature should be about 80 or 90 degrees—then the former degree of 70 or 75 should be slowly resumed as the condition of the case requires.

The patient is confined strictly to the recumbent position, and by the employment of "rubber sheeting," during the application of the hot or cold water, *the risk of shock or chill* (which is very liable to occur if the patient is removed to a hot or a cold bath) *is averted.*

The treatment advocated has been attended by such extraordinary success in the author's hands that he feels justified in calling the attention of the profession to it.

PNEUMONITIS COMPLICATED WITH ILEO-COLITIS.

*Summer Case*—In order to illustrate the applicability of the above treatment in cases occurring in very young infants. the following little case is offered, which occurred at a time of sudden but short lived drop in temperature during a term of intense heat, 98 to 101 degrees, of August.

An infant, age 9 months, was taken ill with pneumonitis (severe cough, dyspnoea, crepitant, and subcrepitant rales), complicated with ileo-colitis (frequently bloody evacuations), pulse 120, respiration 40, temperature 104, semi-comatose, marked restlessness, nausea, and congestion of the conjunctiva.

*Treatment*—Was given—(two hours after the initial dose of hydrarg. chlor. mit. gr. 6) one-third of a powder of formula No. 2 every two hours, with shaved ice and glycerine.

The head and nape of neck, hands and feet were kept thoroughly and constantly wet with "ice water," continuously "fanned."

A *cold, wet pack* was applied to the *praecordium and abdomen.*

She was nourished at the breast.

For the intestinal inflammation, as the tympanites, was given quiniae sulph. gr. 4, glycerinae 23, aqua bulliens ʒIII,—M. ft. enem. Signa.: For intestinal irrigation night and morning. As soon as the temperature was decidedly lower, the ice was removed from the water, and water of the same temperature as was being applied to the praecordium was continued until the "crisis" was reached.

The treatment was equally valuable for both infections. The "crisis" was gradually and permanently impelled within 72 hours. The case was discharged at the fourth visit, no trace of either disease being left.

I learned afterward that she had an uninterrupted convalescence.

